# Comparison of laparoscopic vs. robotic sentinel lymph node mapping and biopsy in  endometrial cancer

**DOI:** 10.1007/s11701-025-02300-w

**Published:** 2025-04-24

**Authors:** Harrison Odgers, Angela Lin, Trevor Tejada-Berges

**Affiliations:** 1 Department of Gynaecologic Oncology, Chris O’Brien Lifehouse, 119-143 Missenden Road, Camperdown, Sydney, 2050 Australia; 2 School of Medicine, The University of Sydney, Sydney, Australia

**Keywords:** Endometrial cancer, Sentinel lymph node biopsy, Robotic surgery, Gynaecologic Oncology, Minimally invasive surgery

## Abstract

We compare the success of sentinel lymph node (SLN) biopsy between standard laparoscopy and robotic-assisted laparoscopy (RAL) in patients with endometrial cancer. Patients with uterine epithelial tumour types undergoing staging surgery were identified from January 2019 to March 2023. Included patients underwent a total hysterectomy, bilateral salpingo-oophorectomy and attempted bilateral SLN biopsy with indigocyanine green (ICG) dye, utilising either standard laparoscopy or RAL. 298 patients met the inclusion criteria. 211 (70.8%) had standard laparoscopy and 87 (29.2%) underwent RAL. The RAL cohort had significantly higher median body mass index (BMI) compared to standard laparoscopy (37 vs. 28 kg/m^2^, *p* < 0.001). The overall rate of successful bilateral SLN biopsy was 66.8% (*n* = 199), and at least one hemi-pelvis was successfully biopsied in 87.3% (*n* = 260) of patients. There was no significant difference in bilateral SLN biopsy success between RAL and standard laparoscopy (60.9% vs 69.2%, *p* = 0.17). RAL was not predictive of bilateral SLN biopsy success in multivariate analysis (OR 1.10, *p* = 0.76). There was no difference in SLN biopsy location, number of nodes identified, or empty-packet dissections between the surgical approaches. Increasing age (OR 0.96, *p* = 0.002) and BMI (OR 0.94, *p* < 0.001) were significantly associated with reduced bilateral SLN biopsy success. Between the learning and experienced periods of the study, the bilateral SLN biopsy success rate improved significantly for RAL (40.6 vs. 72.7%, *p* = 0.03), which was not found with standard laparoscopy. The decision to perform RAL should consider multiple factors including surgeon experience. Future research should be directed towards prospective, randomised and BMI-matched cohorts.

**Supplementary Information:**

The online version contains supplementary material available at 10.1007/s11701-025-02300-w.

## Introduction

Endometrial cancer is the second most common gynaecological malignancy globally, with an estimated 420,242 new diagnoses in 2022 [1]. Rising incidence is often attributed to the obesity epidemic and ageing populations, with the highest rates found in high-income countries [2, 3].

For patients with endometrial carcinomas clinically confined to the uterus, sentinel lymph node (SLN) biopsy has become standard of care [4, 5]. Prospective evidence has shown that a systematic surgical approach, use of indigo-cyanine green (ICG) dye and ultra-staging produces high sensitivity and low false negative rates when compared to systematic lymphadenectomy; with no adverse effect on oncological outcomes [4–8]. In the event of failed SLN mapping, side-specific pelvic/low para-aortic lymphadenectomy can be considered, taking into account uterine risk factors for nodal disease and patient co-morbidities [4, 5].

Robotic-assisted laparoscopy (RAL) is increasingly utilised in Gynae-Oncology, particularly in North America and Europe. RAL differs from standard laparoscopy through the use of a surgical console to control the robotic platform, endoscopic wristed instruments, and 3D vision, all of which may increase surgical accuracy [9]. However, it may be associated with longer operating-room utilisation and increased cost [9, 10]. The benefit of RAL compared to standard laparoscopy for endometrial cancer patients has been described in peri-operative outcomes such as reduced conversion to laparotomy, particularly in those with a body mass index (BMI) ≥ 30 kg/m^2^, however evidence is largely retrospective [11, 12]. There is a paucity of data comparing success rates of SLN biopsy between RAL and standard laparoscopy for endometrial cancer patients [13, 14]. It remains unclear if RAL can be recommended to improve SLN biopsy mapping success.

The primary objective of this study was to compare bilateral SLN biopsy success rate in patients undergoing RAL versus standard-laparoscopy in endometrial cancer staging surgery. To our knowledge, this study represents the second largest data-set investigating this specific clinical question. The secondary objectives were to compare overall (bilateral and unilateral) SLN biopsy success rate, number of SLN per biopsy sample, location of biopsies, empty packet dissections (samples absent of nodal tissue) and metastases identified by SLN biopsy. We also aimed to identify variables affecting bilateral SLN biopsy success, such as tumour characteristics, age and BMI.

## Methods

A retrospective review at an Australian tertiary referral centre between January 2019 and March 2023 was performed. Ethics approval was given by the Sydney Local Health District Ethics Review Committee (X23-0009 and 2022/ETH02757). Participants included were ≥ 18 years, diagnosed with uterine epithelial tumours, and were undergoing staging surgery with either RAL or standard laparoscopy. The surgery involved a total hysterectomy, bilateral salpingo-oophorectomy and bilateral SLN biopsy, with or without infra-colic omentectomy. Those who had previous hysterectomy, fertility-sparing surgery or neo-adjuvant treatment were excluded, as were patients with mesenchymal tumour types. Patients who had ICG injected with no attempt at sidewall dissection were excluded (e.g., unexpectedly advanced disease). The beginning of the study period was chosen to coincide with our centre’s uptake of the SLN biopsy procedure for all patients with apparent uterine confined endometrial cancers. We divided the study period into a ‘learning period’ (years 2019–2020) and ‘experienced period’ (years 2021–2023) for analysis. Oncological staging was as per FIGO 2009 criteria and SLN metastasis size described as per WHO guidelines [15–17]. All five surgeons at our centre are Certified Gynaecologic Oncologists with more than 10 year’s experience at the time of initial data collection. Individual surgeon case-load experience with RAL or standard laparoscopy were not available.

The SLN biopsy approach across surgeons is as follows: ICG at a concentration of 1.25 mg/ml (Verdye^®^ powder, diluted with sterile water) injected into the cervix at 3 and 9 o’clock; 1 mL superficial and 1 mL at a depth of 0.5–1 cm. Injection preceded pelvic sidewall dissection by 15–30 min. After establishment of pneumoperitoneum and survey of the abdomen/pelvis, the pelvic retroperitoneal spaces are opened. Near-infrared overlay is used to identify the lowest echelon node. Suspicious nodes were removed regardless of mapping. Nodes were sent for pathological assessment via standard H&E and ultra-staging with IHC for AE1/AE3 [18]. The DaVinci Xi with Firefly ICG overlay (Intuitive, California, USA) and Stryker 1688 Advanced Imaging Modalities 4 K with ICG overlay (Stryker, Michigan, USA) were used in all cases. Our approach is in line with internationally endorsed expert guidelines [19]. The use of RAL or standard laparoscopy was dependent upon the patient’s BMI, surgeon preference and platform availability.

For this analysis, the success of SLN biopsy procedure was defined as the intra-operative identification of presumed SLN, per-hemi-pelvis. The failure was when either no SLN could be located for removal within a hemi-pelvis, or when the presumed SLN biopsy was absent of nodal tissue on histopathology (empty packet dissection). Lymph nodes removed due to their macroscopic suspicious appearance were not taken to represent a successful SLN biopsy procedure in isolation. Continuous variables were described by medians and categorical variables by percentages. Comparison between groups was performed with Chi-squared or Mann–Whitney *U* test where appropriate. Binary logistic regression was used for univariate and multivariate analysis. All reported *p*-values are two-sided and *p* < 0.05 was considered statistically significant. Data were stored securely via an institutional REDCap database and analysed with SPSS version 29 (IBM, New York, USA). In accordance with the journal’s guidelines, we will provide our data for independent analysis for the purposes of additional data analysis or for the reproducibility of this study in other centres if requested.

## Results

Over the study period, 436 patients with a uterine malignancy had operations at our centre; 298 (68.3%) met inclusion criteria. The clinical and pathological characteristics of the cohort are outlined in Table 1. The median age was 66 years (range 35–87) and median BMI 30.4 kg/m^2^ (range 17–65). Standard laparoscopy was more frequently used (*n* = 211, 70.8%) compared to RAL (*n* = 87, 29.2%). Across all patients, the success rate of bilateral SLN biopsy was 66.8% (*n* = 199), and bilateral failure occurred in 12.7% of patients (*n* = 38). Unilateral success occurred in 20.5% (*n* = 61) of procedures, resulting in at least one hemi-pelvis successfully biopsied in 87.3% (*n* = 260) of all patients. A total of 487 presumed SLN biopsies were sent for histopathological assessment, with 28 (5.8%) of these demonstrating no nodal tissue. The external iliac lymph-node was most frequently biopsied (*n* = 265, 57.7%), followed by the obturator node (*n* = 105, 22.9%). Across 260 patients with at least one hemi-pelvis successfully biopsied, metastases were identified in 30 (11.5%) patients, with six (2.3%) of these being ITCs.

**Table 1:** Characteristics of entire cohort

	*N* = 298 (range, %)
Age (years)	66.0 (35–87)
BMI (kg/m^2^)	30.4 (17–65)
Surgical approach	
Standard laparoscopy	211 (70.8%)
Robotic-assisted laparoscopy	87 (29.2%)
ECOG score	
0	233 (78.2%)
1	47 (15.8%)
≥ 2	18 (6%)
Histology	
Endometrioid	237 (79.5%)
Non-endometrioid	61 (20.5%)
Serous	36 (12.1%)
Clear cell	8 (2.7%)
Carcinosarcoma	13 (4.4%)
Dedifferentiated	4 (1.3%)
Grade (FIGO)	
1	168 (56.4%)
2	45 (15.1%)
3/High grade^a^	83 (27.9%)
LVSI	
Absent	197 (66.1%)
Present	101 (33.9%)
Depth of myometrial invasion	
None	73 (24.5%)
< 50%	102 (34.2%)
≥ 50%	123 (41.3%)
Cervical stromal involvement	
No	268 (89.9%)
Yes	30 (10.1%)
FIGO 2009 Stage	
IA	160 (53.7%)
IB	80 (26.8%)
II	20 (6.7%)
IIIA	9 (3.0%)
IIIB	1 (0.3%)
IIIC1	23 (7.7%)
IVA	2 (0.7%)
IVB	3 (1.0%)
Surgery period	
Learning (2019–2020)	127 (42.6%)
Experienced (2021–2023)	171 (57.4%)
SLN-detection	
Bilateral success	199 (66.8%)
Unilateral success	61 (20.5%)
Bilateral failure	38 (12.8%)
Hemi-pelvises mapped^b^	459 (77%)
Number of SLN identified per mapped hemi-pelvis^c^	
1	342 (74.5%)
2	85 (18.5%)
≥ 3	32 (7%)
Mapping location^c^	
External iliac	265 (57.7%)
Obturator	105 (22.9%)
Common iliac	28 (6.1%)
Internal iliac	11 (2.4%)
Pre-sacral	6 (1.3%)
Para-aortic	3 (0.7%)
Not documented	41 (8.9%)
Lymph node metastases identified by SLN biopsy^d^	30 (11.5%)
ITC	6 (2.3%)
Micrometastasis	8 (3.1%)
Macrometastasis	16 (6.1%)
Empty packet biopsy^e^	28 (5.8%)

*BMI* Body Mass Index, *ECOG* Eastern Cooperative Oncology Group, *LVSI* Lymphovascular space invasion, *FIGO* The International Federation of Gynecology and Obstetrics, *SLN* sentinel lymph node, *ITC* Isolated tumour cells

^a^Includes grade 3 endometrioid and any non-endometrioid epithelial histology

^b^459 out of 596 possible hemi-pelvises

^c^Number of lymph nodes confirmed per biopsy sample. Percentages calculated based on 459 successfully mapped sentinel nodes

^d^Percentages calculated based on 260 patients with at least one SLN successfully biopsied

^e^Percentages calculated based on 487 biopsies sent for histopathological assessment

When comparing RAL to standard laparoscopy, there was no significant difference in the rate of bilateral SLN biopsy success between the two approaches (60.9 vs. 69.2%, *p* = 0.17) [Table 2]. When comparing at least one hemi-pelvis successfully biopsied via SLN (bilateral + unilateral success), there was significantly lower success in those undergoing RAL compared to standard-laparoscopy (79.3 vs. 90.5% vs, *p* = 0.008). Empty-packet dissections were similar between surgical approaches (3.2 vs. 6.6% vs. *p* = 0.15) and there was no significant difference in mapping location of SLN or number of SLN identified per mapped hemi-pelvis (Table 2). The proportion of patients who had metastases identified by SLN biopsy were similar (10 vs. 10.3%, *p* = 0.92). Patients undergoing RAL had a significantly higher median BMI (37 vs. 28 kg/m^2^, *p* < 0.001), were more likely to be classified as endometrioid histotype (87.4 vs. 76.3%, *p* = 0.032) and low grade (FIGO grade1/2) (81.6 vs. 67.3%, *p* = 0.013) compared to those undergoing standard laparoscopy (Table 2).

**Table 2:** Clinical, pathological and sentinel lymph node biopsy characteristics by mode of surgery

	Standard laparoscopy (*n* = 211, 337 biopsied hemi-pelvises) (range, %)	Robotic-assisted laparoscopy (*n* = 87, 122 biopsied hemi-pelvises) (range, %)	*p*-value
SLN biopsy success			
Bilateral success	146 (69.2%)	53 (60.9%)	0.17
Bilateral + unilateral success	191 (90.5%)	69 (79.3%)	0.008
Unilateral success	45 (21.3%)	16 (18.4%)	
Bilateral failure	20 (9.5%)	18 (20.7%)	
Site of mapping of SLN^a^			
External iliac	184 (54.6%)	81 (66.4%)	0.20
Obturator	77 (22.8%)	28 (23.0%)	
Common iliac	23 (6.8%)	5 (4.1%)	
Internal iliac	10 (3.0%)	1 (0.8%)	
Pre-sacral	6 (1.8%)	0 (0%)	
Para-aortic	3 (0.9%)	0 (0%)	
Not documented	34 (10.1%)	7 (5.7%)	
Number of SLN biopsy identified per hemi-pelvis^a^			
1	254 (75.4%)	88 (72.1%)	0.57
2	62 (18.4%)	23 (18.9%)	
≥ 3	21 (6.2%)	11 (9.0%)	
Empty packet^b^			
Yes	24 (6.6%)	4 (3.2%)	0.15
No	337 (93.4%)	122 (96.8%)	
Age (years)			
	67 (35–87)	65 (41–85)	0.30
BMI (kg/m^2^)			
	28 (17–52)	37 (22–65)	< 0.001
ECOG score			
0	171 (81.0%)	62 (71.3%)	0.063
≥ 1	40 (19%)	25 (28.7%)	
Histopathology			
Endometorioid	161 (76.3%)	76 (87.4%)	0.032
Non-endometrioid	50 (23.7%)	11 (12.6%)	
Grade			
Grade 1	105 (49.8%)	63 (72.4%)	0.002
Grade 2	37 (17.5%)	8 (9.2%)	
Grade 3/High grade histology^c^	69 (32.7%)	16 (18.4%)	
LVSI			
Absent	136 (64.5%)	61 (70.1%)	0.35
Present	75 (35.5%)	26 (29.9%)	
Depth of myometrial invasion			
None	50 (23.7%)	23 (26.4%)	0.59
< 50%	76 (36.0%)	26 (29.9%)	
≥ 50%	85 (40.3%)	38 (43.7%)	
Cervical stromal involvement			
No	188 (89.1%)	80 (92.0%)	0.46
Yes	23 (10.9%)	7 (8.0%)	
FIGO 2009 Stage			
IA	106 (50.2%)	54 (62.1%)	0.580
IB	59 (28.0%)	21 (24.1%)	
II	16 (7.6%)	4 (4.6%)	
IIIA	8 (3.8%)	1 (1.1%)	
IIIB	1 (0.5%)	0 (0%)	
IIIC1	18 (8.5%)	5 (5.7%)	
IVA	1 (0.5%)	1 (1.1%)	
IVB	2 (0.9%)	1 (1.1%)	
Surgery period			
Learning (2019–2020)	95 (45%)	32 (36.8%)	0.19
Experienced (2021–2023)	116 (55%)	55 (63.2%)	
Metastases identified by SLN^d^			
No	190 (90.0%)	78 (89.7%)	0.92
Yes	21 (10%)	9 (10.3%)	
ITC Micrometastasis Macrometastasis	3 (1.4%) 6 (2.8%) 12 (5.7%)	3 (3.4%) 2 (2.3%) 4 (4.6%)	

*SLN* = Sentinel Lymph Node, *BMI* = Body Mass Index, *ECOG* Eastern Cooperative Oncology Group, *LVSI* Lymphovascular space invasion, *FIGO* The International Federation of Gynecology and Obstetrics, *ITC* Isolated tumour cells

^a^Percentages calculated based on hemi-pelvis number for column

^b^Percentages calculated based on 487 biopsies sent for histopathological assessment

^c^Include grade 3 endometrioid and any non-endometrioid histology

^d^Percentages calculated based on 260 patients with at least one SLN successfully biopsied

Variables associated with bilateral SLN biopsy success are outlined in Table 3. Increasing BMI (33 vs. 29.4 kg/m^2^ *p* < 0.001) and age (67 vs. 65 years, *p* = 0.011) were significantly associated with failure, as was performance status; defined by the Eastern Cooperative Oncology Group (ECOG) score, ≥ 1 vs. 0 (*p* = 0.027). There was no overall significant difference in bilateral SLN biopsy success between the ‘learning’ and ‘experienced’ periods (61.4 vs. 73.8%, *p* = 0.09). When stratified by surgical approach, the success rate of bilateral SLN biopsy via RAL significantly increased between the learning and experienced periods (40.6 vs. 72.7%, *p* = 0.003). The success of SLN biopsy via standard laparoscopy was unchanged (68.4 vs. 69.8%, *p* = 0.83) (Supplementary Material 1). There was no significant difference in the rate of bilateral SLN biopsy success between surgical approaches in those with BMI ≥ 30 kg/m^2^ and age ≥ 65 years (Supplementary Material 2).

**Table 3:** Clinical, pathological and sentinel lymph node mapping characteristics by sentinel lymph node biopsy success

	Bilateral SLN biopsy success (*N* = 199) (range, %)	Unilateral or bilateral SLN biopsy failure (*N* = 99) (range, %)	*p*-value
BMI (kg/m^2^)			< 0.001
	29.4 (17–56)	33 (19–65)	
Age (years)			0.011
	65 (35–87)	67 (47–87	
Surgery period			0.09
2019–2020	78 (61.4%)	49 (38.6%)	
2021–2023	121 (70.8%)	50 (29.2%)	
ECOG			0.027
0	163 (70%)	70 (30%)	
≥ 1	36 (55.4%)	29 (44.6%)	
Histopathology			0.19
Endometrioid	154 (65%)	83 (35%)	
Non-endometrioid	45 (73.8%)	26.2%)	
Depth of myometrial invasion			0.32
None	53 (72.6%)	20 (27.4%)	
< 50%	63 (61.8%)	39 (38.2%)	
≥ 50%	83 (67.5%)	40 (32.5%)	
LVSI			0.89
None	131 (66.5%)	66 (33.5%)	
Present	68 (67.3%)	33 (32.7%)	
Cervical stromal involvement			0.23
No	176 (65.7%)	92 (34.3%)	
Yes	23 (76.7%)	7 (23.3%)	
Grade			0.86
1	110 (65.5%)	58 (34.5%)	
2	31 (68.9%)	14 (31.1%)	
3/High-grade^a^	58 (68.2%)	27 (31.8%)	

*SLN* Sentinel Lymph Node, *BMI* Body Mass Index, *ECOG* Eastern Cooperative Oncology Group, *LVSI* Lymphovascular space invasion

^a^Includes grade 3 endometrioid and any non-endometrioid histology

In multivariate regression analysis, RAL was not a significant predictor of bilateral SLN biopsy success (OR 1.10, *p* = 0.76). Across the entire cohort, both increasing age (OR 0.96, *p* = 0.002) and BMI (OR 0.94, *p* < 0.001) were significantly associated with a reduction in bilateral SLN biopsy success. Surgery performed in the experienced period had a trend towards predicting bilateral SLN biopsy success across the entire cohort, however this was non-significant (OR 1.56, *p* = 0.091) (Table 4).

**Table 4:** Univariate and multivariate binomial regression analysis predicting bilateral sentinel lymph node biopsy success

	Bilateral SLN biopsy success, *N* = 199 (range, %)	Bilateral or unilateral SLN biopsy failure, *N* = 99 (range, %)	Univariate analysis		Multivariable analysis	
			OR (95% CI)	*p*-value	OR (95% CI)	*p*-value
Age (years)	65 (35–87)	67 (47–87)	0.97 (0.94–0.99)	0.004	0.96 (0.933–0.99)	0.002
BMI (kg/m^2^)	29.4 (17–56)	33 (19–65)	0.947 (0.922–0.974)	< 0.001	0.94 (0.91–0.97)	< 0.001
ECOG score						
≥ 1	36 (18.1%)	29 (29.3%)	0.53 (0.30–0.94)	0.029	0.88 (0.48–1.64)	0.70
0	163 (81.9%	70 (70.7%)	–	–	–	–
Year of surgery						
Experienced (2021–2023)	121 (60.8%)	50 (50.5%)	1.52 (0.94–2.47)	0.091	1.56 (0.93–2.56)	0.091
Learning period (2019–2020)	78 (39.2%)	49 (49.5%)				
Mode of surgery						
Robotic	53 (26.6%)	34 (34.3%)	0.70 (0.41–1.17)	0.17	1.10 (0.60–2.04)	0.76
Laparoscopic	146 (73.4%)	65 (65.7%)	–	–	–	–
Histopathology						
Non-endometrioid	45 (22.6%)	16 (16.2%)	1.52 (0.81–2.85)	0.20		
Endometrioid	154 (77.4%)	83 (83.8%)				
Cervical stromal involvement						
Yes	23 (11.6%)	7 (7.1%)	1.72 (0.71–4.15)	0.23		
No	176 (88.4%)	92 (92.9%)	–	–		
LVSI						
Present	68 (34.2%)	33 (33.3%)	1.04 (0.62–1.73)	0.89		
Absent	131 (65.8%)	66 (66.7%)	–	–		
Grade						
1	110 (55.3%)	58 (58.6%)	–	–		
2	31 (15.6%)	14 (14.1%)	1.17 (0.59–2.37)	0.67		
3/High grade^a^	58 (29.1%)	27 (27.3%)	1.13 (0.65–1.98)	0.66		
Depth of myometrial invasion						
None	53 (26.6%)	20 (20.2%)	–	–		
< 50%	63 31.7%)	39 (39.4%)	0.61 (0.32–1.17)	0.14		
≥ 50%	83 (41.7%)	40 (40.4%)	0.78 (0.41–1.48)	0.45		

*Odds ratio is to predict success of bilateral SLN biopsy*

*SLN* Sentinel Lymph Node, *BMI* Body Mass Index, *ECOG* Eastern Cooperative Oncology Group, *LVSI* Lymphovascular space invasion

^a^Grade 3 endometrioid and any non-endometrioid histology

## Discussion

### Summary of main results

Our study found no difference in the primary outcome, rate of bilateral SLN biopsy success, between RAL and standard laparoscopy. RAL was also not a predictor of bilateral SLN biopsy success in multivariate regression analysis. In the secondary outcome of overall SLN biopsy success, bilateral plus unilateral, standard laparoscopic approach demonstrated a significantly better performance compared to RAL. We identified BMI as a significant predictor of bilateral SLN biopsy success across the entire cohort, with a 1-unit increase leading to 4% increased risk of failure. Increasing age was also significant, with a 1-year increase associated with a 6% increased risk of failure. Improvement in bilateral SLN biopsy success between the learning and experienced periods was significant only in the RAL approach. The median BMI of the RAL cohort was significantly higher than those who underwent standard laparoscopy.

### Results in context of the published literature

There are only two previously described studies comparing SLN biopsy success in endometrial cancer between RAL and standard laparoscopy using ICG dye [13, 14]. Our primary findings are similar to the larger Italian single-centre review, which found no significant difference between surgical approaches in bilateral SLN biopsy success despite their RAL cohort having a significantly higher median BMI (26 vs. 34.8 kg/m^2^) [13]. These authors also found a significant improvement in bilateral SLN biopsy success with increasing SLN biopsy experience, however, in contrast to our study this effect was seen across both surgical approaches [13]. The smaller Australian single-centre study also found no difference in bilateral SLN biopsy success between surgical approaches (laparoscopy, 89 vs. RAL, 83.3%), however, in contrast to our study, the median BMI was balanced between groups [14]. A meta-analysis combining these studies found no difference in overall and bilateral SLN biopsy detection rates, number of SLN identified, intra and post-operative complications, and time to complete dissection, however, noted the relative lack of data with only two studies included [20].

In our data, the lowest bilateral SLN biopsy success rates were in the RAL cohort during the learning period, with significant improvement in the experienced period. A learning curve for the SLN biopsy procedure has been described in endometrial cancer patients [13, 21–24]. A study of SLN biopsy with RAL suggested gradual improvement then a plateau of bilateral SLN biopsy success after 40 cases [21]. In our study, a learning curve may have only been seen with the RAL cohort due to our centre having less experience compared to standard laparoscopy. The surgeon experience of RAL differs to standard laparoscopy in several ways including lack of sensory feedback and instrument control, thus a learning curve for new RAL surgeons is expected, and has been described in multiple surgical specialties including Gynaecologic-Oncology [25–28]. The variables used to measure the learning curve in RAL surgery include time-based metrics, complication rates, and in the case of SLN procedure, biopsy success rate [21, 26]. RAL was increasingly available at our centre from the beginning of the study period, coinciding with the increased uptake of the SLN-B procedure. However, we did not collect individual surgeon case number experience in terms of RAL or SLN biopsy procedure as part of this study, therefore, we consider these specific findings hypothesis-generating.

Increasing BMI has been associated with increased risk of SLN biopsy failure in endometrial cancer patients, however the data are conflicting [29–31]. A meta-analysis of predictors of SLN biopsy failure suggested no association with BMI ≥ 30 kg/m^2^, however, a large multi-centre retrospective cohort study, not included in the meta-analysis, found that each 5-unit BMI increment led to a 15.6% increased risk of biopsy failure [30]. Our study similarly demonstrated that increasing BMI led to increased risk of bilateral SLN biopsy failure, consistent across both surgical approaches. Increased BMI may cause this effect via inadequate pelvic side-wall access, difficulty identifying nodal tissue within adiposity, and higher anaesthetic risk leading to a less aggressive surgical approach [21, 29, 30]. At an anatomical level there may be obesity-mediated impairment of pelvic lymphatic function [32]. Our RAL cohort had a significantly higher median BMI than the standard laparoscopy cohort. However, in multivariate analysis controlling for BMI, RAL was not a predictor of SLN biopsy success, and when considering only those with BMI ≥ 30 kg/m^2^, mapping rates were similar between groups (RAL 56.3% vs. standard laparoscopy 59.3%, *p* < 0.70). While our study provides evidence for BMI as a predictor of SLN mapping failure, it does not provide evidence that RAL improves SLN biopsy mapping success compared to standard laparoscopy in those with raised BMI.

Consistent with our findings, increasing age is an independent risk factor for SLN biopsy failure in endometrial cancer patients [33]. A large multi-centre retrospective trial estimated that each 10-year-age increment led to a 28% increase in SLN biopsy failure [33]. This may be due to age-related deterioration of pelvic lymphatic function and surgeon and/or patient preference for less extensive staging in older patients due to concerns over increased morbidity [33, 34]. A further mechanism could be the increased prevalence of non-endometrioid histology and LVSI in the older cohort, given both pathological factors have been linked with reduced SLN detection [33, 35]. Our results failed to demonstrate any histopathological predictor of SLN biopsy success. This correlation with age was independent of surgical approach.

### Strengths and weaknesses

To our knowledge, this is the second largest cohort amongst handful of studies comparing SLN biopsy success rates between RAL and standard laparoscopy in endometrial cancer; thus expanding the knowledge of an under-investigated clinical question. Our study included important metrics such as SLN biopsy location, number of biopsied nodes, and rate of empty packet dissection. Complete data were available for all patients, with histopathology uniformly described via synoptic pathological reports and BMI routinely collected at our pre-operative clinic. We acknowledge several limitations. Data on individual surgeon experience of the RAL and SLN biopsy procedures were not collected, therefore, despite the possible learning-curve trend observed, we are unable to specifically describe the effect of individual surgeon experience on SLN biopsy success. Data on complication rates, anaesthetic time and cost were not collected, and we acknowledge these are important in deciding surgical approach. We did not collect data regarding patient factors and surgeon intra-operative impression of contributors to non-mapping of SLN (e.g., obesity, pelvic adhesions, uterine size, previous surgery, endometriosis). Recording these factors may have helped clarify reasons for mapping failure. Intra-operative impression of challenging SLN dissection in more significantly obese patients, thus surgeons perusing a less aggressive approach, may have contributed to the lower mapping rates in the RAL cohort. Due to the retrospective nature of the study design, we are unable to confirm if the SLN biopsy procedure was technically performed in the same way across all patients. This may affect the generalizability of results; however, we also acknowledge that use of a SLN biopsy algorithm does not guarantee removal of the true SLN. Finally, the outcomes of interest such as empty-packet rate were uncommon, limiting our conclusions of the effect of surgical approach.

### Implications for practice and future research

Prospective randomised data are required to satisfactorily answer this clinical question. The retrospective cohort studies generally have unbalanced arms with higher median BMI in the robotic cohorts, as with our study. Prospective trials should include agreed upon standardised approach to SLN biopsy and include information on surgeon experience, complication rates, time to complete the procedure, intra-operative impressions of contributors to non-mapping, and cost. The currently recruiting RObese trial has some of these criteria [36]. This prospective randomised multi-centre European trial will compare RAL to standard laparoscopy in endometrial cancer patients with BMI > 30 kg/m^2^ [36]. The primary endpoint is conversion to laparotomy. Secondary endpoints listed in the study protocol do not include SLN biopsy mapping, however, this end-point could form a sub-analysis. The RObese trial is expected to complete recruitment in 2026.

## Conclusions

There was no difference in the rate of bilateral SLN biopsy success between RAL and standard laparoscopy despite patients undergoing RAL having a significantly higher BMI. In our cohort, increasing BMI and age are risk factors for failed bilateral SLN biopsy. Lowest bilateral SLN mapping rates were seen when both RAL and the SLN biopsy were new to our centre. Surgeons should factor their experience and patient characteristics when considering surgical approach. Prospective randomised data are required to determine the benefit of the different MIS in SLN biopsy success rates, particularly given it has become standard in many centres.

## Supplementary Information

Below is the link to the electronic supplementary material.

Supplementary file1 (PDF 58 KB)

## Data Availability

Anonymised data that support the findings of this study are available from the corresponding author upon reasonable request.
