# Supplementary material for: Comparison of laparoscopic vs. robotic sentinel lymph node mapping and biopsy in endometrial cancer
Source: J Robot Surg. 2025 Apr 24;19(1):173. doi: 10.1007/s11701-025-02300-w (PMC12021943; doi:10.1007/s11701-025-02300-w)
Supplement: Supplementary file 1 — Supplementary file1 (PDF 58 KB) [file 11701_2025_2300_MOESM1_ESM.pdf]

Supplementary Material 1: Sentinel lymph node biopsy success by surgery period stratified by surgery type.

Surgery type	Surgery period	Bilateral SLN biopsy success (n=199)	Unilateral or bilateral SLN biopsy failure (n=99)	p-value
Standard laparoscopy (n=211)	Learning period[1] (n=95)	65 (68.4%)	30 (31.6%)	0.83
	Experienced period[2] (n=116)	81 (69.8%)	35 (30.2%)	
Robotic-assisted Laparoscopy (n=87)	Learning period (n=32)	13 (40.6%)	19 (59.4%)	0.003
	Experienced period (n=55)	40 (72.7%)	15 (27.3%)	

SLN = Sentinel Lymph Node.
1: Learning period: All patients who had operations in the calendar years 2019-2020
2: Experienced period: All patients had operations in the calendar years years 2021-2023

Supplementary Material 2: Sentinel lymph node biopsy success by surgical approach stratified by body-mass index and age categories.

Stratification Category	Surgical approach	Bilateral SLN biopsy success (n=199)	Unilateral or bilateral SLN biopsy failure (n=99)	p-value
BMI				
< 30 kg/m^2	Laparoscopic (n=120)	92 (76.7%)	28 (23.3%)	0.68
	Robotic (n=16)	13 (81.3%)	3 (18.8%)	
≥ 30 kg/m^2	Laparoscopic (n=91)	54 (59.3%)	37 (40.7%)	0.70
	Robotic (n=71)	40 (56.3%)	31 (43.7%)	
Age				
< 65 years	Laparoscopic (n= 87	67 (77.0%)	20 (23.0%)	0.51
	Robotic (n=41)	29 (70.7%)	12 (29.3%)	
≥ 65 years	Laparoscopic (n=124)	79 (63.7%)	45 (36.3%)	0.22
	Robotic (n=46)	24 (52.2%)	22 (47.8%)	

BMI = Body Mass Index. SLN = Sentinel Lymph Node.
